# Correction to “Understanding Temperature Profiles of Distillation Columns”

**DOI:** 10.1021/acs.iecr.4c01893

**Published:** 2024-05-30

**Authors:** Lena-Marie Ränger, Ivar J. Halvorsen, Thomas Grützner, Sigurd Skogestad

The recent publication contains seven rules to explain the appearance of temperature pinches in a distillation column separating a relative ideal mixture into two products. For binary mixtures, the pinches will appear either at the column end (for pure products, Rule 1) or next to the feed stage (Rule 4). However, when applying the proposed rules to a more complex dividing-wall (Petlyuk) column, we realized that the rules may be a bit unclear. We therefore give some additional explanations on the use the rules (Figure 1) and provide two revised rules (Table 1).

**Table 1:** Proposed Pinch Rules

Rule 1	A constant temperature zone (pinch zone) at a column end (top or bottom) indicates an almost pure component in the corresponding product. This pinch zone is observable independently of the total number of stages.
Rule 2	The top and bottom sections can each have a maximum of two pinch zones at the same time. The appearance of two pinches in the same section means that the section is operated at minimum energy. If only one pinch is visible in a section, the product is either over- or underpurified.
Rule 3	At the boundaries between neighboring operating regions, i.e., along the minimum energy lines, the locations of pinch zones are the same as in the adjacent regions.
Rule 4	There is an “invariant” pinch temperature when operating at minimum energy or less, which does not change when varying the operating point within the given region of the diagram. The invariant pinch can be observed in the upper part (e.g., if all components are in the bottom product) or the lower part of the column. If all components are present in both product flows, the invariant pinch is above and below the feed stage and has the feed boiling temperature.
**Rule 5 (revised)**	A pinch only on one side of the feed stage means that all components are present in the product stream at the corresponding column end. In this case, the pinch temperature does not equal the feed boiling point. If this pinch appears in combination with another pinch (rules 2 and 3), one component is at the limit to appear but not actually present (minimum energy case).
**Rule 6 (revised)**	A pinch in the middle of a section indicates that at least two but not all feed components are present in the corresponding product. If this pinch appears in combination with another pinch (rules 2 and 3), one component is at the limit to appear but not actually present (minimum energy case).
Rule 7	A clearly visible pinch in one section in combination with a poorly visible one in the other section is an indication for a nonoptimal feed stage.

**Figure 1:**
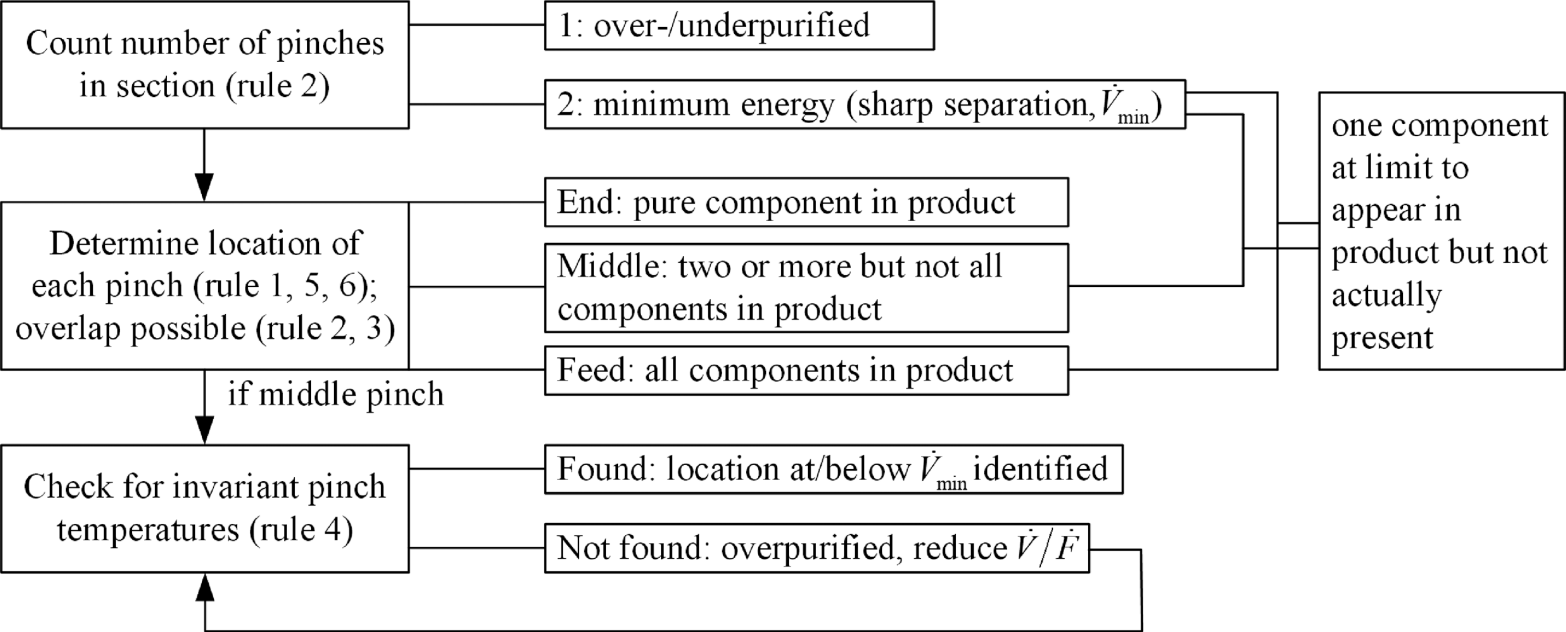
Flowchart for how to use the rules in Table 1.

For nonbinary mixtures (with 3 components or more), there can be pinches in the middle of a section. Rule 6 was meant to describe a middle pinch, and based on the excellent work of King^1,2^ (Chapter 7; 1971, page 361; 1980, page 334), the term “the feed pinch moves” was chosen. However, this phrasing may be misleading, because using the theory of Underwood they have a different mathematical origin. Also, the original Rule 6 was potentially a bit misleading at it did not include King’s assumption that it applied to nonkey components. To rectify this, we propose the following restatement of Rule 6:

**Revised Rule 6**. A pinch in the middle of a section indicates that at least two but not all feed components are present in the corresponding product. If this pinch appears in combination with another pinch (rules 2 and 3), one component is at the limit to appear but not actually present (minimum energy case).

Similarly, we propose the following extended Rule 5:

**Revised Rule 5**. A pinch only on one side of the feed stage means that all components are present in the product stream at the corresponding column end. In this case, the pinch temperature does not equal the feed boiling point. If this pinch appears in combination with another pinch (rules 2 and 3), one component is at the limit to appear but not actually present (minimum energy case).

The complete set of rules is given in Table 1.

As an example, the temperature profile for a sharp A–BC separation in Figure 9(a) of the original paper is reprinted in Figure 2 here. The heavy component C appears in the bottom only. In both the top and bottom section, there are two pinches, so the column is operated at minimum energy (rule 2).

**Figure 2:**
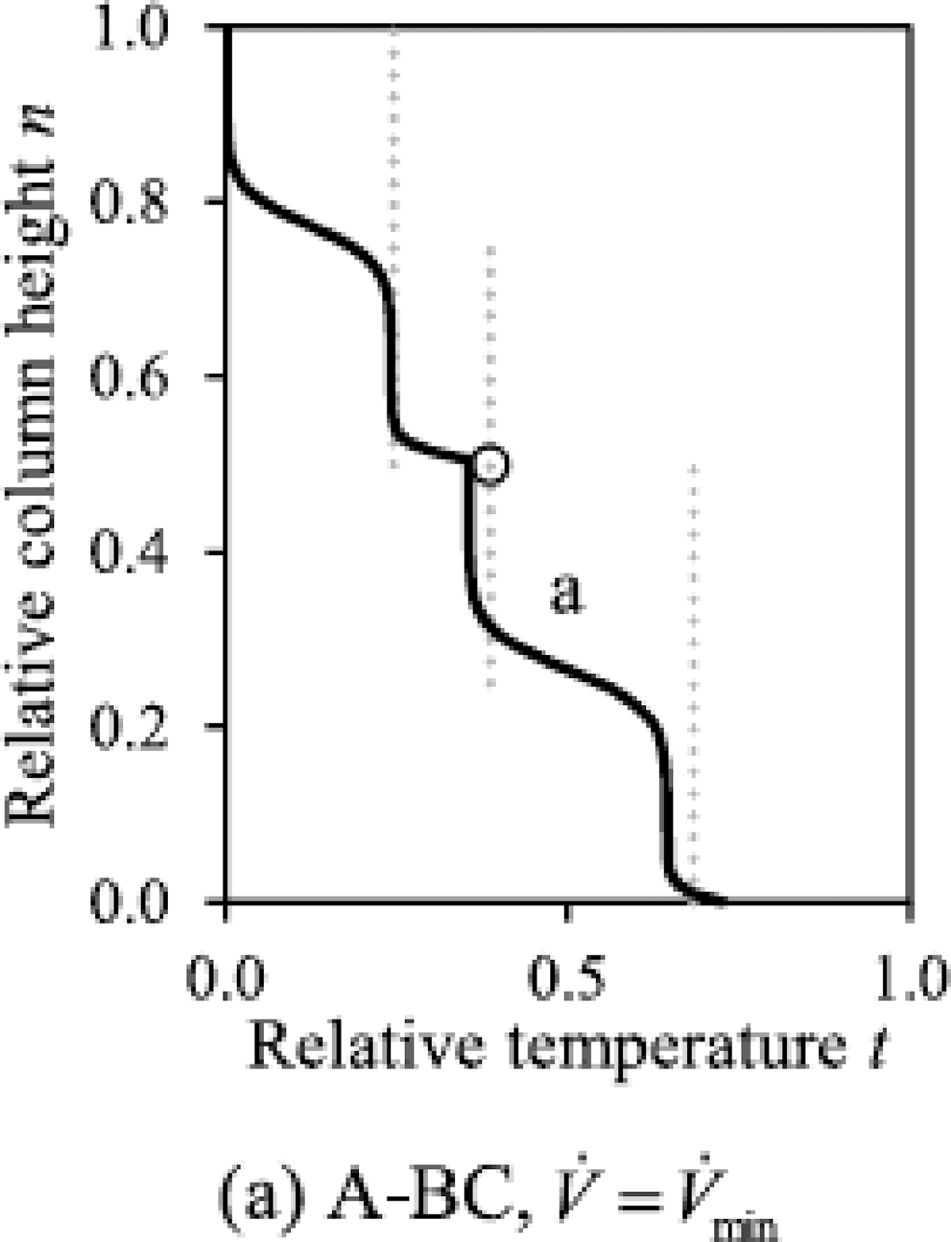
Temperature profile for minimum energy operation (large number of stages) for sharp A–BC separation for ternary ABC mixture.

For the top section there is one pinch at the top end, meaning the top product is pure A (rule 1). The second pinch is in the middle, which means that two but not all components are present in the product (revised rule 6). The two components present in the top are A and B, which may seem a bit strange since we just stated that the top product is pure A. However, component B is at the limit to appear at minimum energy operation (rule 2).

For the bottom section, there is a pinch directly below the feed stage and one in the middle (although quite close to the bottom). The feed pinch in combination with a second one means that we are at the limit to obtain all components present in the bottom product; however, one component does not actually appear, which has to be the light boiler A in this case (revised rule 5).

At this point, we already know that the bottom product consists of components B and C. However, if the analysis would start with the middle pinch, we would end up with the same result: Based on the first part of the revised rule 6, the middle pinch means that two or more but not all feed components are present. For the ternary feed this means that B and C are in the bottom product. As this pinch appears in combination with a second pinch, one component is about to appear in the bottom product. The second pinch is below the feed stage, and rule 5 says that all components are about to appear in the product. Correspondingly, the component that is at the limit but actually not present is component A.
